# B cell phenotype and serum levels of interferons, BAFF, and APRIL in multisystem inflammatory syndrome in children associated with COVID-19 (MIS-C)

**DOI:** 10.1186/s40348-023-00169-z

**Published:** 2023-10-28

**Authors:** Adam Klocperk, Marketa Bloomfield, Zuzana Parackova, Ludovic Aillot, Jiri Fremuth, Lumir Sasek, Jan David, Filip Fencl, Aneta Skotnicova, Katerina Rejlova, Martin Magner, Ondrej Hrusak, Anna Sediva

**Affiliations:** 1 Department of Immunology, 2nd Faculty of Medicine, Charles University and University Hospital in Motol, V Uvalu 84, 150 06, Prague, Czech Republic; 2 Department of Paediatrics, 1st Faculty of Medicine, Charles University and Thomayer University Hospital, Prague, Czech Republic; 3 Institute of Organic Chemistry and Biochemistry of the Czech Academy of Sciences, IOCB Gilead Research Center, Prague, Czech Republic; 4 Department of Paediatrics - PICU, Faculty of Medicine in Pilsen, Charles University, Pilsen, Czech Republic; 5 Department of Paediatrics, 2nd Faculty of Medicine, Charles University and University Hospital in Motol, Prague, Czech Republic; 6 Department of Pediatric Hematology, CLIP - Childhood Leukaemia Investigation Prague, 2nd Faculty of Medicine, Charles University and University Hospital in Motol, Prague, Czech Republic; 7 Department of Paediatrics and Inherited Metabolic Disorders, First Faculty of Medicine, Charles University and General University Hospital in Prague, Prague, Czech Republic

**Keywords:** PIMS-TS, MIS-C, COVID-19, Interferon, BAFF, APRIL, SLE

## Abstract

**Background:**

Multisystem inflammatory syndrome in children associated with COVID-19 (MIS-C) is a late complication of pediatric COVID-19, which follows weeks after the original SARS-CoV-2 infection, regardless of its severity. It is characterized by hyperinflammation, neutrophilia, lymphopenia, and activation of T cells with elevated IFN-γ. Observing the production of autoantibodies and parallels with systemic autoimmune disorders, such as systemic lupus erythematodes (SLE), we explored B cell phenotype and serum levels of type I, II, and III interferons, as well as the cytokines BAFF and APRIL in a cohort of MIS-C patients and healthy children after COVID-19.

**Results:**

We documented a significant elevation of IFN-γ, but not IFN-α and IFN-λ in MIS-C patients. BAFF was elevated in MIS-C patient sera and accompanied by decreased BAFFR expression on all B cell subtypes. The proportion of plasmablasts was significantly lower in patients compared to healthy post-COVID children. We noted the pre-IVIG presence of ENA Ro60 autoantibodies in 4/35 tested MIS-C patients.

**Conclusions:**

Our work shows the involvement of humoral immunity in MIS-C and hints at parallels with the pathophysiology of SLE, with autoreactive B cells driven towards autoantibody production by elevated BAFF.

**Graphical Abstract:**

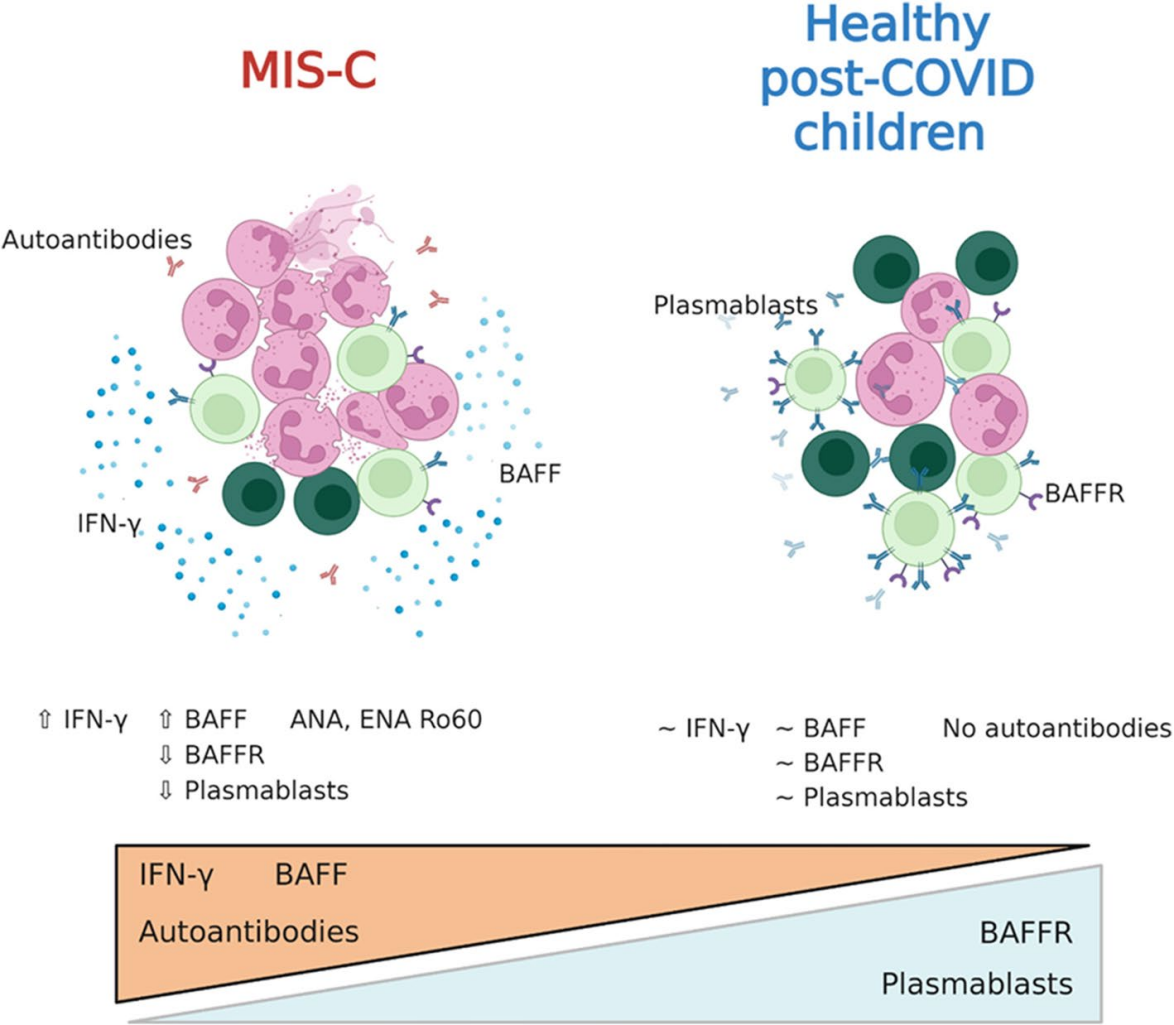

## Background

Multisystem inflammatory syndrome in children associated with COVID-19 (MIS-C) is now a well-specified entity described in a number of excellent publications that map in detail the immune/autoimmune/inflammatory responses accompanying this condition [1–5]. Clinically, the hallmark symptoms include fever, rash, conjunctivitis, mucositis and serositis, lymphadenopathy, gastrointestinal, respiratory, cardiovascular, and neurocognitive symptoms, which develop on the background of markedly increased acute phase reactants and inflammatory markers in temporal association with SARS-CoV2 infection in genetically susceptible individuals. This pro-inflammatory state is accompanied by alterations in cellular populations of innate and acquired immunity, with prominent lymphopenia during the acute stage of the disease, which typically lags several weeks after acute SARS-CoV-2 infection, regardless of its severity. The lymphopenia is characterized by a decrease in T cells, but at the same time with their activation and clonal proliferation [1]. Significant alterations were shown in T cell subpopulations in MIS-C, and recent reports also show their clonality and exhaustion [2, 3]. Importantly, a number of publications document the presence of autoimmune phenomena and autoantibodies in MIS-C patients, targeting both systemic and tissue- or organ-specific antigens consistent with the systemic nature of the disease, yet also associated with its characteristic clinical presentation in specific organs, mainly the heart [4, 5]. These findings suggest a strong polyclonal antibody response driven by activated B cells. B cells, however, are less studied in the context of MIS-C. B cell counts were reported normal or decreased, in line with the general MIS-C-associated lymphopenia [6, 7]. Some studies have shown an increase in plasmablasts, as well as IgD^−^CD27^−^ double negative B cells [8, 9]. Similar IgD and CD27 double-negative activated B cells were previously documented in systemic lupus erythematosus (SLE) in association with disease activity and autoantibody secretion [4], pointing to a certain parallel in B cell activation and autoantibody production between systemic autoimmune diseases such as SLE and MIS-C. In SLE, this polyclonal autoantibody production is driven by the serum cytokine B cell activating factor (BAFF) and a proliferation-inducing ligand (APRIL) [5, 10]; however, this association has not yet been studied in MIS-C.

Similarly, while a dysregulated IFN-γ response is also a feature shared between MIS-C and SLE [11–14], the activity of type I and type III interferons has so far only been shown in SLE and rare inborn autoinflammatory disorders [15, 16], but not in MIS-C, even though antibodies against type I interferons have been shown to contribute to COVID-19 mortality and severity [17].

To explore these immune factors contributing to the hyperinflammation in MIS-C, we set out to assess type I, II, and III interferons, serum BAFF, APRIL, and B cell phenotype and BAFFR expression in children with acute MIS-C and in healthy children after COVID-19.

## Patients and methods

### Patients and controls

The MIS-C cohort was recruited from patients admitted to the Department of Pediatrics, University Hospital in Motol, Prague, Department of Pediatrics, Thomayer University Hospital, Prague, and Department of Pediatrics, University Hospital in Pilsen, Pilsen, Czech Republic. Informed consent with participation in this study was signed by the participants’ legal guardians in accordance with the Declaration of Helsinki, and the study was approved by the Ethical Committee of the University Hospital in Motol, reference no. EK-1376/21. Data on demographics, clinical manifestations, routine laboratory features, and other investigations, therapeutic management, and outcomes were collected retrospectively from the medical records of the patient or obtained via patient/parent interview.

In MIS-C patients, samples were obtained through peripheral venepuncture after patient admission, before administration of corticosteroids or immunoglobulins. Patients were included in the study based on their MIS-C diagnosis consistent with WHO criteria [18]. In total, 50 MIS-C patients were recruited during the inclusion period between October 2020 and April 2021, 24 females, aged 11 months to 18 years (7.8 ± 4.35 years, mean ± SD). The alpha (B.1.1.7) SARS-CoV-2 variant was dominant in Czechia during this period.

As a control cohort, 7 healthy children who previously underwent COVID-19, 2 female, aged 1 to 14 years (9.9 ± 3.9 years), were recruited into the study (hereafter referred to as healthy post-COVID children). These healthy donors were sampled 4–6 weeks after their SARS-CoV-2 PCR positivity.

For assessment of BAFF and APRIL, 4 MIS-C patients, 2 females, aged 1.4 to 5.5 years (3.5 ± 1.4 years), were re-evaluated 6 months after discharge from hospital (hereafter referred to as MIS-C convalescent). Further, 8 healthy donor children with no history of COVID-19, 5 females, aged 11.6 to 17.2 years (13.7 ± 1.9 years), were included for comparison (hereafter referred to as healthy children).

The description of cohorts is summarized in Table 1.

**Table 1:** Cohort characteristics

	***n***	**Sex**	**Age years range (mean ± SD)**
MIS-C	50	24 females	0.9–18 (7.8 ± 4.35)
MIS-C convalescent	4	2 females	1.4–5.5 (3.5 ± 1.4)
HD children post-COVID	7	2 females	1–14 (9.9 ± 3.9)
HD children	8	5 females	11.6–17.2 (13.7 ± 1.9)

### Flow cytometry

For evaluation of peripheral blood B cell phenotype, the blood was taken into ethylenediaminetetraacetic acid (EDTA)-coated tubes as described above. PBMCs were obtained using Ficoll-Paque (Pharmacia, Uppsala, Sweden) and cryopreserved in liquid nitrogen.

After thawing, PBMCs were incubated in the presence of recombinant human DNAse I (Pulmozyme, Roche, Prague, Czechia; final concentration was 10 IU/mL) in complete media (RPMI 1640 supplemented with 10% of heat-inactivated fetal calf serum, penicillin (50 U/mL), streptomycin (50 U/mL) and 1.7 mM sodium glutamate) for 30 min at 37 °C in a CO_2_ incubator. One million cells were resuspended in 100 μL phosphate-buffered saline (PBS) (Sigma-Aldrich, St. Louis, MO) and stained for 30 min in the dark at room temperature with CD5 BV421 (Cat No. 562646, BD Biosciences, San Jose, CA), IgM BV510 (Cat No. 314522, Biolegend, San Diego, CA), BAFFR BV711 (Cat No. 743573, BD Biosciences), and a dried mixture of IgD FITC, CD27 PE, CD24 PerCP-Cy5.5, CD19 PE-Cy7, CD21 APC, and CD38 APC-Cy7 (Custom-design dry reagent tube, Exbio Praha, Vestec, Czechia). Then, 2 mL of BD FACS™ Lysing Solution (BD Biosciences) was added and cells were incubated for 10 min in the dark, room temperature. In the end, cells were washed once in PBS with 1% BSA and pellets were resuspended in 150 μL PBS.

Flow cytometry measurement was performed on BD FACSLyrics (BD Immunocytometry Systems, San Jose, CA). FlowJo software was used for data analysis (TreeStar, Ashland, OR).

### ELISA

For evaluation of serum cytokine levels, the blood was taken into uncoated tubes as described above, and serum was separated by centrifugation and stored frozen at − 80 °C until further evaluation. BAFF and APRIL were measured according to the manufacturer’s specifications using pre-made ELISA kits (BAFF from R&D Systems, Minneapolis, USA, APRIL from Abcam, Cambridge, UK). Type I, II, and III interferons (specifically, pan-IFN-α, IFN-γ, and IFN-λ1) were quantified following the manufacturer’s protocols of Human ELISA Basic KIT (HRP) (MABTECH, Sweden) using Nunc MaxiSorp flat-bottom 96-well plates (Invitrogen). An absorbance of 450 nm was read by multimode plate reader EnVision 2105 (PerkinElmer).

### Statistics

Statistical analysis was performed using Brown-Forsythe and Welch one-way analysis of variance (ANOVA) and unpaired *t* tests with Welch’s correction in GraphPad Prism 8.0 (San Diego, CA, USA). Values of *p* = 0.01–0.05 (*), *p* = 0.001–0.01 (**), *p* < 0.001 (***), and *p* < 0.0001 (****) were considered statistically significant.

## Results

**Fig. 1:**
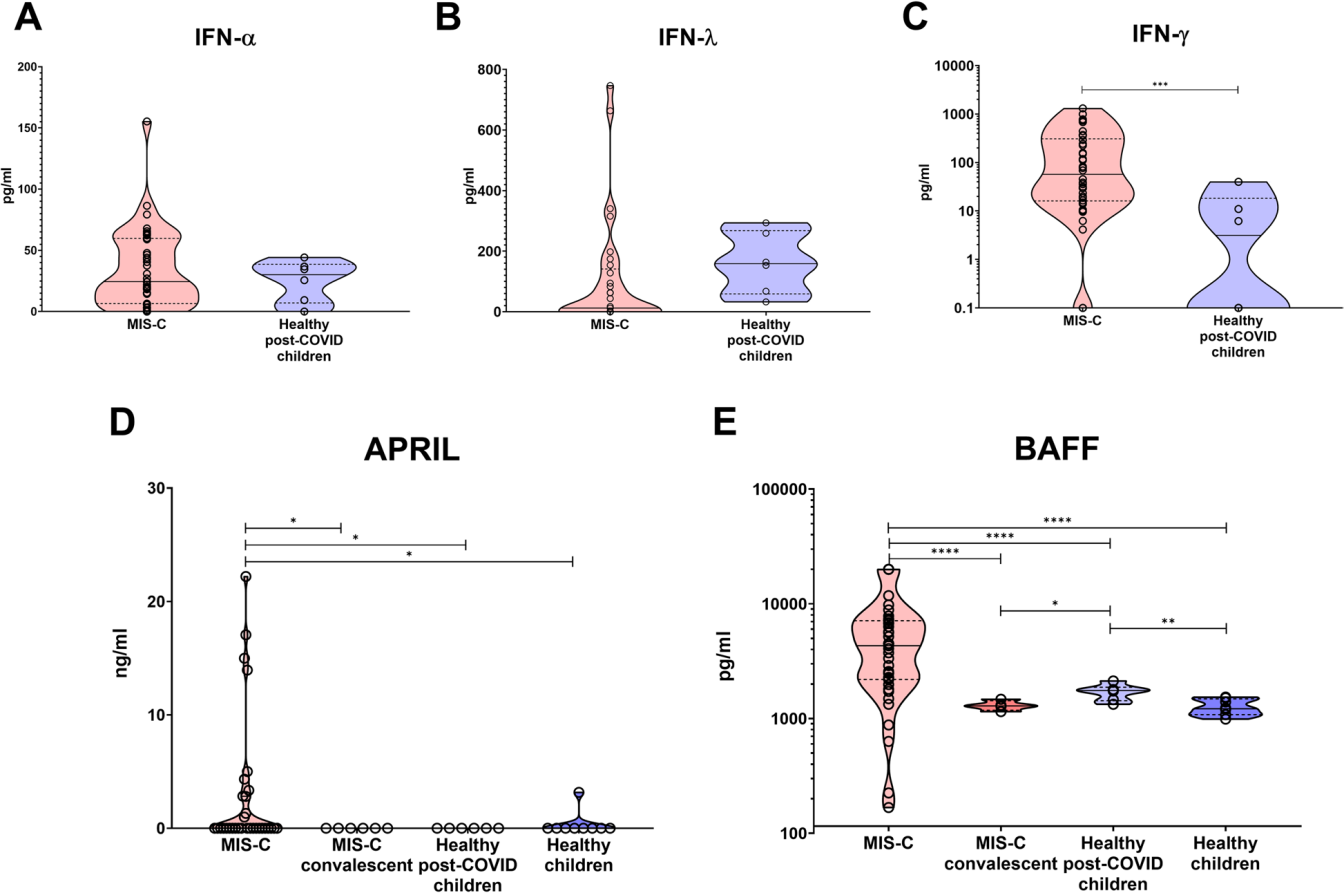
Cytokines in MIS-C patients. **A** IFN-α, **B** IFN-λ, and **C** IFN-γ in MIS-C patients and healthy donor children 6 weeks after COVID-19. **D** APRIL and **E** BAFF in MIS-C patients, convalescent MIS-C patients 6 months after disease resolution, healthy donor children 6 weeks after COVID-19, and healthy donor children with no history of COVID-19 or MIS-C

To analyze the hyperinflammatory signature of MIS-C, we measured IFN-α, IFN-γ, and IFN-λ in the serum of 50 patients with MIS-C sampled shortly after admission to hospital, before administration of immunosuppressive therapy, and compared them to the sera of healthy children who underwent COVID-19 cca 6 weeks prior to sampling and had no signs of MIS-C. We saw no significant changes in IFN-α (*t* test with Welch’s correction *p* = 0.27) and IFN-λ levels (*p* = 0.33) (Fig. 1A, B), but IFN-γ was significantly elevated in MIS-C compared to healthy post-COVID children (*p* = 0.0004) (Fig. 1C).

Next, we measured the presence of autoantibodies and serum concentration of BAFF and APRIL, two cytokines supporting the development and survival of B cells, which are also implicated in other systemic autoimmune diseases reminiscent of MIS-C, such as SLE, systemic vasculitides, or Kawasaki disease [19–21]. Of 35 patients in whom autoantibodies were tested, 4/35 (11%) had positive Ro60 antibodies, 1/35 (3%) had autoantibodies against the Sm antigen, and further, 4/35 (11%) had positive extractable nuclear antigen (ENA) screening. Serum levels of APRIL were largely below the assay detection limit, although in a subset of MIS-C patients we detected elevated APRIL levels, which were missing in post-COVID children, healthy children with no history of COVID-19 or MIS-C, and even in convalescent MIS-C patients sampled several months after full recovery (Fig. 1D). The clinical course of the disease was not distinctly different in these 4 children with high APRIL, and nevertheless, the majority of samples tested APRIL-negative, and as such, the differences were not significant.

Serum BAFF levels, on the other hand, varied significantly between acute MIS-C, convalescent MIS-C patients, post-COVID healthy children, and children with no history of COVID-19 or MIS-C (Brown-Forsythe ANOVA, *p* < 0.0001) (Fig. 1E). Acute MIS-C patients had the highest BAFF levels of all cohorts, which in particular were higher than those in post-COVID children (*t* test with Welch’s correction, *p* < 0.0001), but also than those in convalescent post-MIS-C patients (*p* < 0.0001). Interestingly, even healthy post-COVID children had elevated serum BAFF levels compared to healthy children without a history of COVID-19 (*p* = 0.0093). In both MIS-C patients and healthy children, the trend remained identical, with higher BAFF during/after disease, and lower after full recovery or in times of full health.

**Fig. 2:**
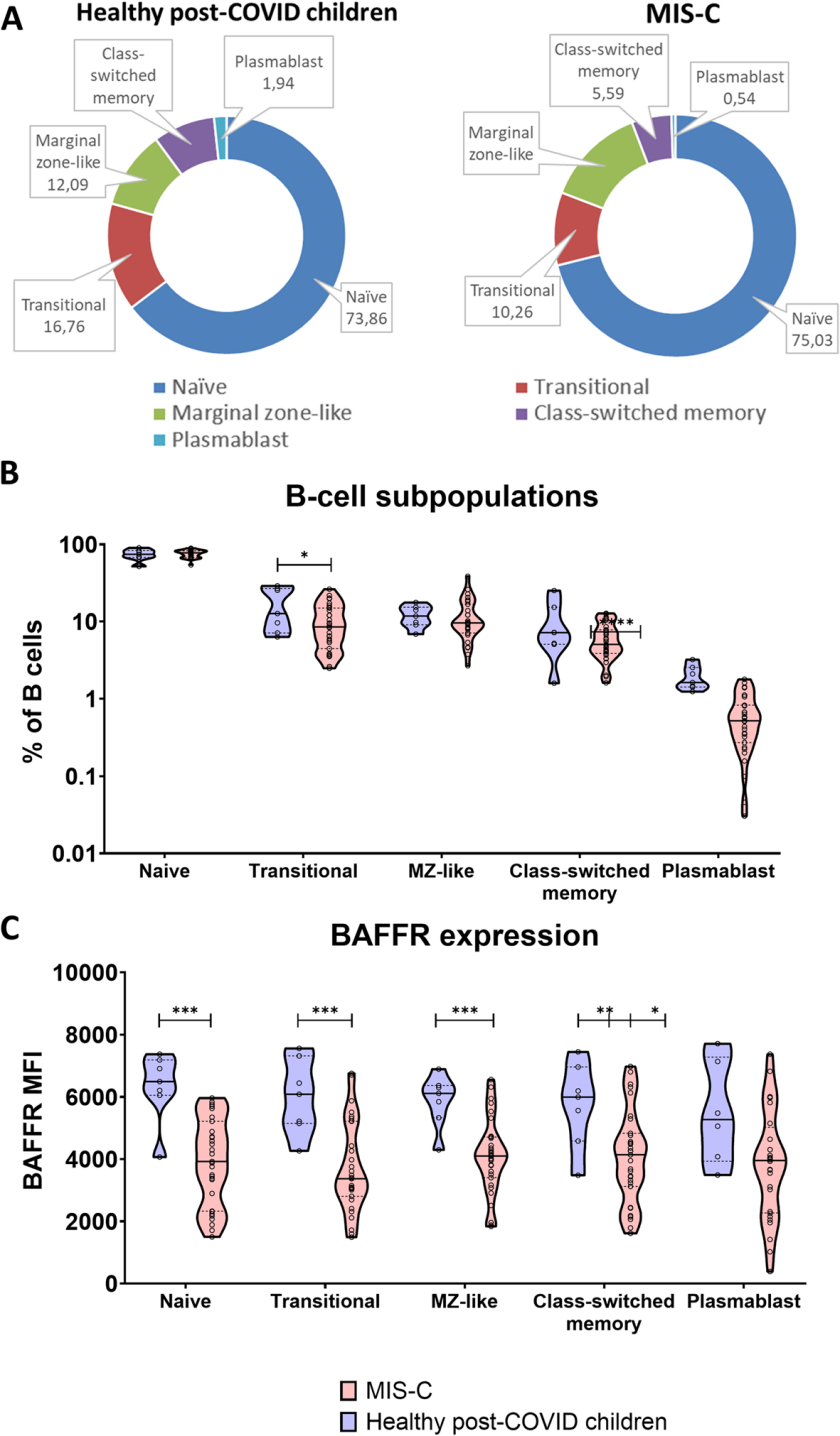
B cell phenotype in MIS-C patients. **A**, **B** B cell subpopulations in MIS-C patients and healthy donor children 6 weeks after COVID-19. **C** BAFFR expression in naive, transitional, MZ-like, switched memory, and plasmablast B cells of MIS-C patients and healthy donor children 6 weeks after COVID-19

Finally, to assess the impact of upregulated BAFF signaling on B cell development, we performed B cell subpopulation phenotyping in patients and healthy donors. We noted a highly significant decrease of circulating plasmablasts (*p* < 0.0001), less significant decrease of transitional B cells (*p* = 0.029), and a slight but insignificant expansion of naïve B cells in MIS-C patients (Fig. 2A, B). Additionally, all MIS-C B cells had strikingly suppressed BAFF receptor (BAFFR) expression (Fig. 2C). This was true in all B cell subsets, including the naïve and transitional subsets.

## Discussion

In this study, we explored the role of type I and II interferons and the dysregulation of humoral immunity in MIS-C, drawing on parallels with other autoimmune diseases, such as SLE.

Our results point towards comparable type I interferon response between acute MIS-C and post-COVID-19 children without MIS-C, but demonstrate increased levels of type II interferon (IFN-γ) between these groups. Type II interferon has been described as a dominant feature of MIS-C previously [11, 12] and may reflect the concurrent T cell activation [1]. However, *B* cell-intrinsic IFNgR signaling has also been shown to evoke spontaneous generation of autoreactive germinal centers and its knock-out results in protection from systemic autoimmunity in animal models [19, 20]. Therefore, the increased IFN-γ levels may represent a contributing mechanism to the autoantibody induction in MIS-C. On the other hand, our data suggests that lingering type I and III interferon inflammation are not robust driving factors behind MIS-C pathogenesis, despite their role in the anti-SARS-CoV-2 immune response [17, 19].

The elevation of IFN-γ and the presence of autoimmune phenomena in MIS-C draws a parallel to SLE, which prompted the exploration of B cell immunity in our patients [13, 20]. Although we did not find any significant differences in the serum levels of the B cell survival-promoting cytokine APRIL between healthy donors, acute, convalescent MIS-C, and post-COVID children, we did observe a stark elevation of BAFF in acute MIS-C children and less so in healthy post-COVID children. Porritt et al. also found increased BAFF mRNA levels in MIS-C children [22]—this significant increase of BAFF levels in MIS-C is consistent with polyclonal B cell activation resulting in the spectrum of detected autoantibodies and clinical manifestations of MIS-C-associated autoimmune systemic and organ inflammation. In our cohort, of 35 patients in whom autoantibodies were tested, 4/35 (11%) had positive Ro60 antibodies and further 4/35 (11%) had positive extractable nuclear antigen (ENA) screening. Previous studies reported the presence of anti-La, a characteristic autoantigen of SLE and Sjogren’s disease, and anti-Jo-1, characteristic of idiopathic inflammatory myopathies, in MIS-C patients [21]. Although later works suggested that these autoantibodies may be derived from high-dose intravenous immunoglobulins [23], administered to the MIS-C patients, our samples were obtained prior to the treatment. The combination of increased IFN-γ and a consequent increase of BAFF has been previously described in SLE, again suggesting an analogy between MIS-C and SLE [24]. Interestingly, in the case of SLE, it has been shown that neutrophils can contribute to an increase in BAFF and a strengthening of the autoimmune process. In the case of MIS-C, hallmarked by marked neutrophilia and disturbed neutrophil functionalities [25, 26], a similar parallel might exist and contribute to the induction of autoimmunity [27].

Finally, we observed a shift in the maturation of B cells, skewing their subpopulations towards antibody-producing plasmablasts and away from the early transitional B cells in MIS-C children. Such reduction of plasmablasts is interesting given their lower reliance on BAFF for survival, which is also bolstered by APRIL [28]. Instead, naïve and transitional B cells, which are highly dependent on BAFF for survival [29], were both decreased in MIS-C children. However, at the same time, all MIS-C B cells had strikingly suppressed BAFF receptor (BAFFR) expression (Fig. 2C)—including naïve and transitional. These results are consistent with the similar situation observed in SLE, where high levels of BAFF are associated with decreased BAFFR expression on all B cell subtypes [30].

In summary, our brief report complements the current understanding of the dysregulation of humoral immunity and autoimmune phenomena seen in MIS-C and highlights the important role of the BAFF-BAFFR axis in their induction.

## Data Availability

The datasets used and/or analyzed during the current study are available from the corresponding author on reasonable request.

## Abbreviations

APRIL: A proliferation-inducing ligand
BAFF: B cell activating factor
COVID-19: Coronavirus disease 2019
HD: Healthy donor
IFN: Interferon
MIS-C: Multisystem inflammatory syndrome in children associated with COVID-19
PBMC: Peripheral blood mononuclear cell
PBS: Hosphate-buffered
SLE: Systemic lupus erythematosus
